# Slicing and support structure generation for 3D printing directly on B-rep models

**DOI:** 10.1186/s42492-019-0013-x

**Published:** 2019-05-22

**Authors:** Kanle Shi, Conghui Cai, Zijian Wu, Junhai Yong

**Affiliations:** 1 School of Software, Tsinghua University, Beijing, China; 2 Beijing National Research Center for Information Science and Technology, Beijing, China; 3 Key Laboratory for Information System Security, Ministry of Education of China, Beijing, China

**Keywords:** 3D printing, Boundary representation model, Slicing, Support structure generation, Intersection curve

## Abstract

Traditional 3D printing is based on stereolithography or standard tessellation language models, which contain many redundant data and have low precision. This paper proposes a slicing and support structure generation algorithm for 3D printing directly on boundary representation (B-rep) models. First, surface slicing is performed by efficiently computing the intersection curves between the faces of the B-rep models and each slicing plane. Then, the normals of the B-rep models are used to detect where the support structures should be located and the support structures are generated. Experimental results show the efficiency and stability of our algorithm.

## Background

Recently, 3D printing has been widely used. However, printing quality and speed issues of 3D printing remain unsolved.

Slicing is a foundational operation of 3D printing. It requires computing the intersection curves of models and slicing planes. This operation is time-consuming, and is a key factor that affects printing quality.

Most slicing algorithms work on standard tessellation language (STL) models [1–4], as STL is a standard file format for 3D printing. However, the data in the STL file format is a discretized form of the 3D models, which contain discretization errors. Thus, many studies considered a slicing algorithm on the original data of the 3D models.

Chen et al. [5] used the AutoSection toolkits provided by PowerSHAPE to directly slice computer aided design (CAD) models. Cao and Miyamoto [6] implemented slicing on the entity models in AutoCAD. Starly et al. [7] performed slicing operations directly on non-uniform rational basis spline models in the standard for the exchange of product model data format. Pandey et al. [8] proposed a slicing procedure for fused deposition modelling (FDM), based on a real-time edge profile of deposited layers.

Support structure generation is another foundational operation of 3D printing. It affects printing quality and material consumption. If the necessary support structures are missed, then 3D printing will fail. In addition, unnecessary support structures mean more required materials and more printing time.

Alexander et al. [9] proposed a method for support generation based on the orientation and size of the patch in the STL model. This method is adopted by several commercial 3D printers, but the support structures that it generates can be reduced, practically.

Some research [10–14] aimed to produce better structures to reduce the quantity of support structure. Wang et al. [11] proposed a support structure consisting of some thin rods. These thin rods are from different directions as compared to a simple vertical direction [9], with much fewer support structures. Chen et al. [5, 15] presented an optimized thin rod structure. The optimized structure has better printability and stability. The thin rods are easier to remove from the surface. Vanek et al. [13] proposed an algorithm to generate a tree-like support structure. This algorithm used thin rods to form a tree-like structure, which is more stable. Dumas et al. [12] presented a scaffolding support structure. This structure has better support strength and stability than the tree-like structure.

The contributions of our study are a slicing algorithm for boundary representation (B-rep) models and a support structure generation algorithm. The slicing algorithm is directly based on B-rep models that reduce the error produced from discretization. The support structure generation algorithm focuses on detecting the hanging edges and points of the model, which will probably appear as draping in printing. The support for the hanging edges and points is to ensure the printing quality of the model.

The remaining parts of this paper are arranged as follows. Section 2 briefly introduces the framework of the algorithm, then it presents the detail of model slicing and support structure generation separately. The experimental results are presented in Section 3. Section 4 provides conclusions for the study.

## Methods

### Overview

**Fig. 1:**
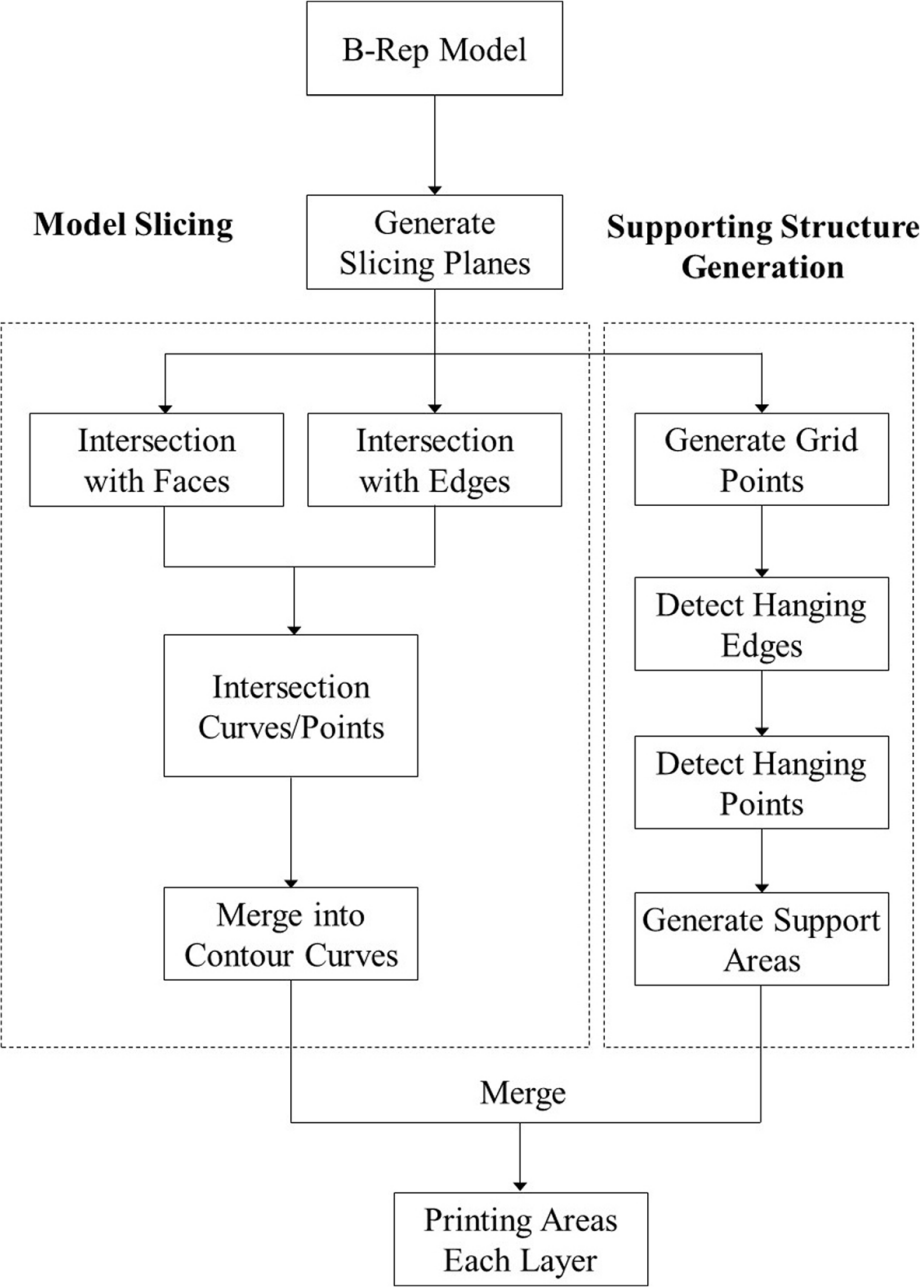
Framework of the algorithm

Model slicing and support structure generation are two key steps in 3D printing. The framework of our slicing and support structure generation algorithm is illustrated in Fig. 1.

First, we calculate all slicing planes according to the size of the B-rep model. Then, we compute the intersection curves between the faces of the B-rep model and each slicing plane, as well as the intersection points between the edges of the B-rep model and each slicing plane. These intersection points are used to split the intersection curves into several segments, where the intersection curves only intersect each other, at most, at endpoints. Thus, the intersection points become endpoints of the intersection curves. Lastly, we analyze the intersection curves to obtain a contour curve of the B-rep model on each slicing plane.

For support structure generation, we compute the grid points of the B-rep model. With the normals of the grid points we compute the supporting region inside, in which hanging points and edges are also detected, to obtain supporting areas for each slicing layer. Lastly, we generate support structures for 3D printing.

### Model slicing

Our slicing operations work directly on B-rep models. The workflow is as follows.

#### Generate slicing planes

We compute the height of the B-rep model along the slicing direction. We generate the slicing planes according to the distance between two neighbor slicing layers.

#### Calculate intersections

We compute the intersection curves between each face in the B-rep model and each slicing plane, as well as the intersection points between each edge in the B-rep model and each slicing plane.

#### Generate contour curve for each layer

We analyze the intersection curves to obtain a contour curve of the B-rep model on each slicing plane.

#### Generate slicing planes

The slicing direction is perpendicular to the slicing planes. We rotate the B-rep model for the Z axis to be in the slicing direction. Then, we calculate the height of the B-rep model along the Z axis. The number of slicing planes is the height of the B-rep model divided by the distance between the two neighbor slicing planes. We set the Z-coordinate of the lowest point of the B-rep model to be 0. All the equations representing slicing planes are obtained as follows:

$$ Z=i\;{d}_{sp}, $$

where *d*_*sp*_ is the distance between the two neighbor slicing planes, *i* = 0, 1, …, $\left\lfloor \frac{h_B}{d_{sp}}\right\rfloor$, *h*_*B*_ is the height of the B-rep model along the Z axis, and ⌊∙⌋ is the floor function, which produces the greatest integer less than the given number. Here, *d*_*sp*_ is given by the user before 3D printing.

#### Calculate intersections

In model slicing, the most difficult step is computing the intersection results between faces or edges of the B-rep model and each slicing plane. In the B-rep model, faces are usually made by trimmed surfaces. Therefore, computing the intersection curves between a face in the B-rep model and a slicing plane can be divided into two steps. First, the intersection curves ***C***_***is***_ are computed between the untrimmed surface of the face and the slicing plane. Second, the intersection curves ***C***_***is***_ are cut by the boundary of the face.

**Fig. 2:**
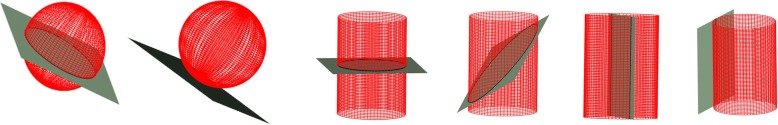
Intersection results depend on directions of surfaces and slicing planes

To speed up computing the intersection curves ***C***_***is***_ between the untrimmed surface and the slicing plane, we develop several intersection functions, each of which works on a type of surface, such as a plane, sphere, ellipsoid, cylinder, elliptical cylinder, cone, and elliptical cone. The resultant intersection curves depend on the directions of surfaces and slicing planes, as illustrated in Fig. 2. The intersection curves ***C***_***is***_ are directly discretized during the process of intersection, and thus are converted into a set of polylines {*P*_*i*_ | *P*_*i*_ = {*p*_*1*_, *p*_*2*_, …, *p*_*n*_}}, where *P*_*i*_ is a polyline, *p*_*k*_ (*k* = 1, 2, …, *n*) is a discrete point, and *n* is the number of discrete points in the polyline *P*_*i*_.

**Fig. 3:**
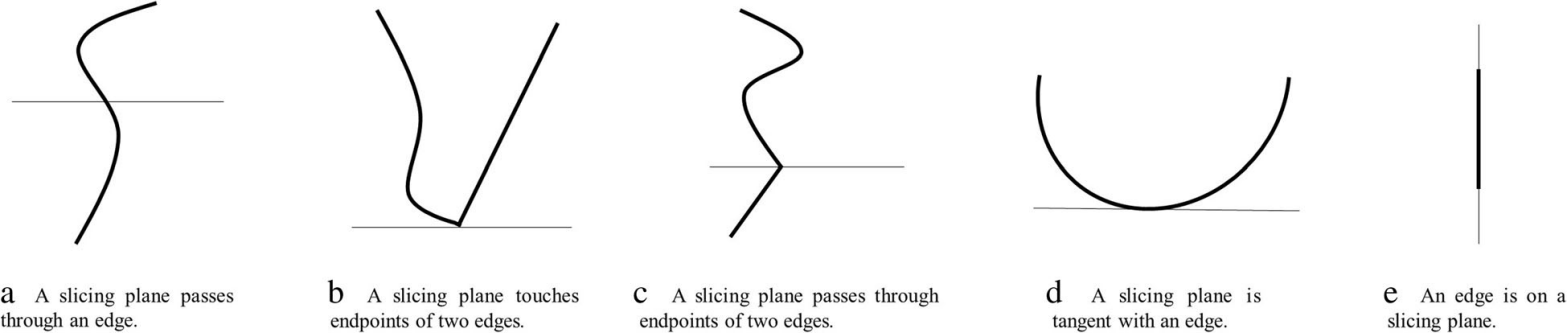
Intersection results depend on directions of edges and slicing planes

Computing the intersection results is a difficult task. Numerous cases exist, and these cases should be considered separately. Figure 3 depicts some cases of computing intersection points between the edges of the B-rep model and slicing planes. The results depend on the directions of edges and slicing planes.

**Fig. 4:**
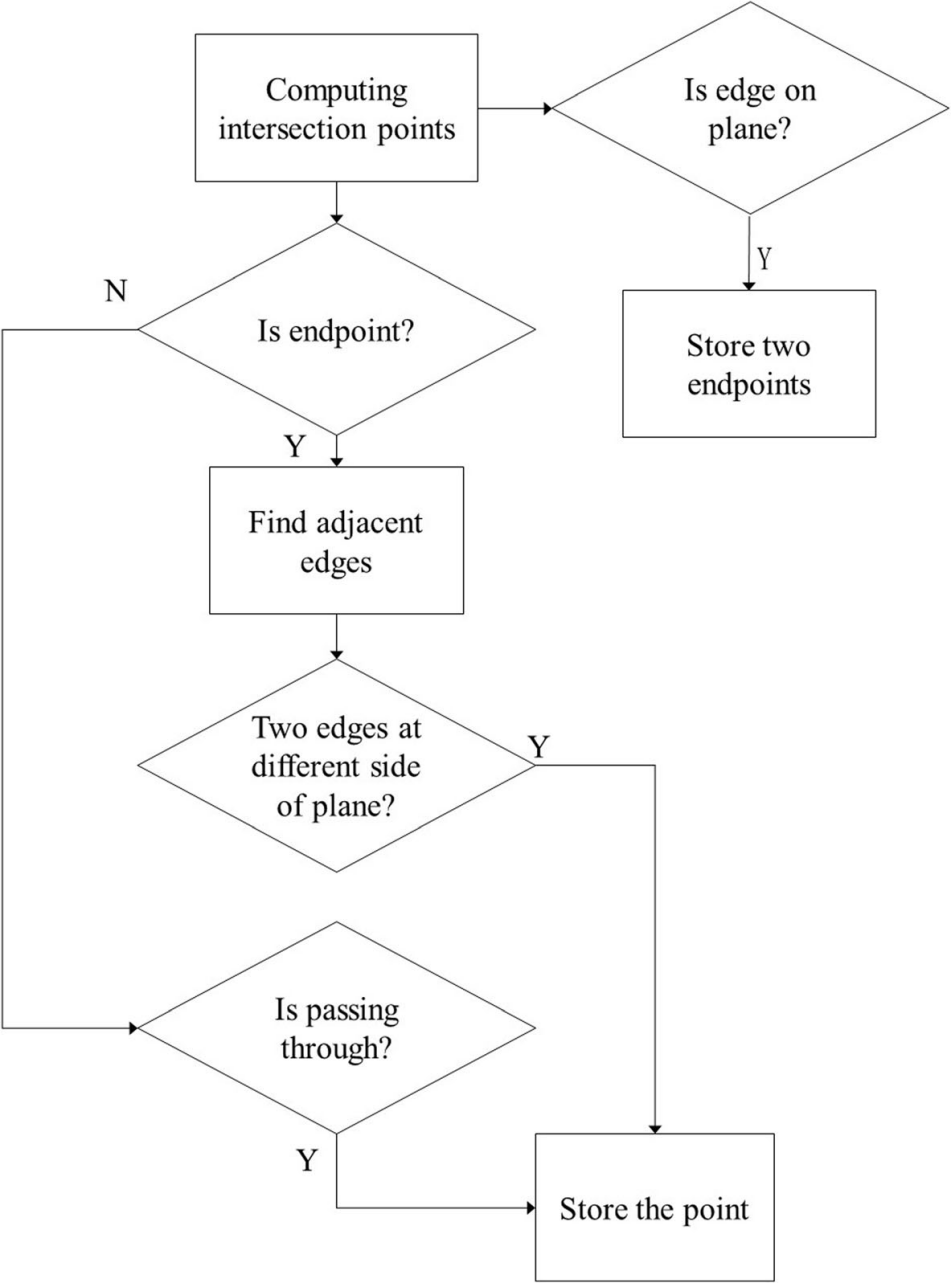
Flow chat of computing intersection points between edges and a slicing plane

The flowchart for computing intersection points between the edges of the B-rep model and a slicing plane is presented in Fig. 4. First, we determine whether an endpoint of the edge is an intersection point. Thus, we find two adjacent edges of the point. If the two adjacent edges are on the same side of the slicing plane (Fig. 3b, then we abandon the intersection point. If the two adjacent edges are on different sides of the slicing plane (Fig. 3c), then we store the intersection point. If the intersection point is on an edge but is not the endpoint of the edge, then the intersection point divides the edge into two segments. If the two segments are on the same side of the slicing plane (Fig. 3d), then we abandon the intersection point. If the two segments are on different sides of the slicing plane (Fig. 3a), then we store the intersection point. If the edge is on the slicing plane (Fig. 3e), then we store two endpoints of the edge.

#### Generate contour curve

Before generating contour curves, we use the intersection points between the edges and each slicing plane to split the intersection curves between the faces and each slicing plane into several segments, such that each intersection curve segment intersects each other segment, at most, at endpoints. An intersection point should be at least on an intersection curve because an edge should be on a face. Thus, the splitting can be performed. We then connect the intersection curve segments of the splitting results to form loops, which are the contour curves on the B-rep model and the slicing planes.

### Support structure generation

For fused-deposition 3D printing, support structure generation is necessary for hanging parts of models. Otherwise, 3D printing may fail because of gravity. Our support structure generation can be performed automatically. The workflow is as follows.

- Generate Grid Points: Grid points are sampled directly on the B-rep models, and the normals, height, and support type information are stored.
- Detect Hanging Edges/Points: Hanging edges and points that may be missing during sampling are detected.
- Generate Supporting Areas: Supporting areas are generated according to the results of the above two steps.

#### Supporting types

In our support structure generation, supporting is classified into the following three types.

- None: Indicates that the grid point has no specific supporting type.
- ForceSupport: Indicates that the grid point is a forced supporting point.
- ForceNoSupport: Indicates that no support can be imposed on the grid point.

The three supporting types can be manually set on some grid points. As support structures may make touch points on models less smooth, it is necessary to manually set the supporting types on some grid points, so as to guarantee the quality of some parts of models.

#### Generate grid points

We create points evenly along the X-axis and Y-axis. Then, we project these points on the B-rep models along the Z-axis to generate the grid points on the B-rep models. These grid points are also the sample points on the B-rep models. We calculate the normals and the height values at the grid points, and store the support types at the grid points.

#### Detect hanging edges and points

**Fig. 5:**
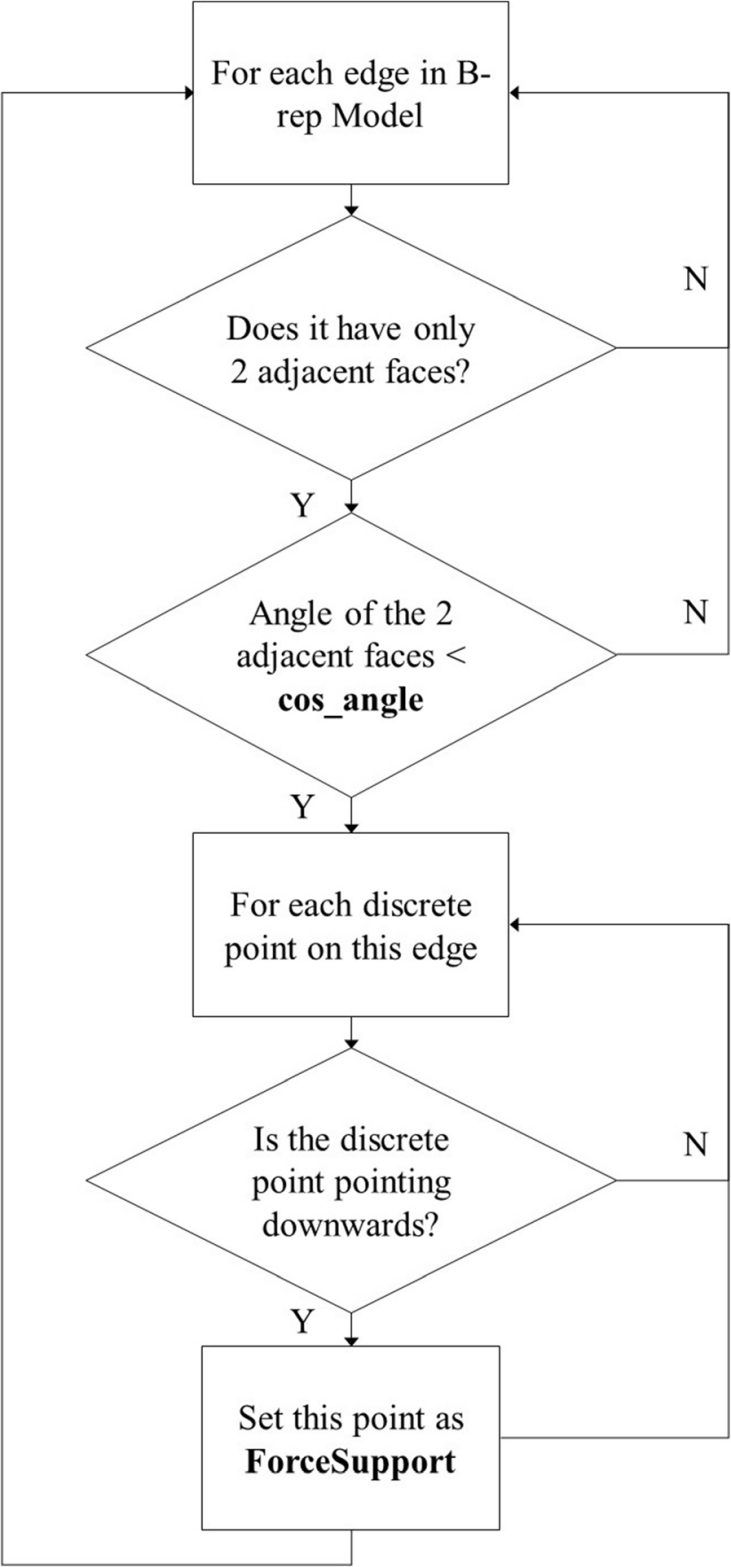
Flow chat of detecting hanging edges

**Fig. 6:**
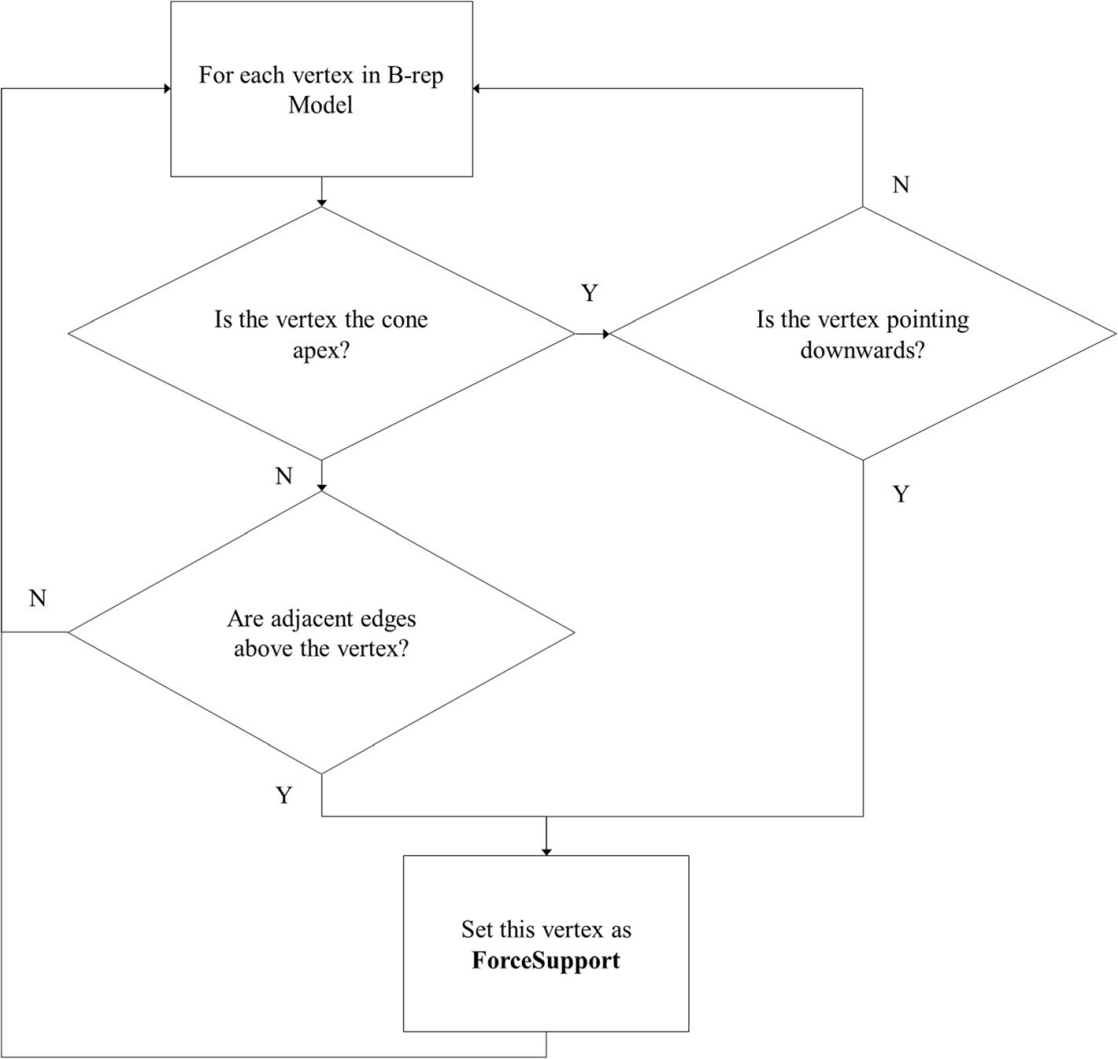
Flow chat of detecting hanging points

Given that edges and vertices are crucial on the B-rep models, they must also be detected. If we find that edges or vertices on the B-rep models are hanging, then we will set them with the supporting type **ForceSupport**. The edges that are hanging are called hanging edges, and the vertices that are hanging are called hanging points. The flowcharts for detecting hanging edges and points are presented in Figs. 5 and 6, respectively.

As shown in Fig. 5, we judge whether an edge has 2 adjacent faces. If the edge has 2 adjacent faces, we judge whether the angle of these adjacent faces is smaller than a threshold **cos_angle**. Then, we discretize the edge and judge whether the discrete point is pointing downwards. We calculate the angle between the normal of the discrete point and the vector (0, 0, − 1). If the angle is smaller than **cos_angle**, we consider the point as pointing downwards, and mark the point as **ForceSupport**. The value of **cos_angle** is π/3.

As shown in Fig. 6, there are two kinds of vertices which are hanging points. The first one is the cone apex. If the vertex is the cone apex and the vertex is pointing downwards, we mark this vertex as **ForceSupport**. The algorithm for determining whether the vertex is pointing downwards is described above. The second kind of vertex, which is a hanging point, is a vertex in which all of its adjacent edges are above it. We calculate all the endpoints of its adjacent edges, and if the *z* values of these endpoints are not smaller than the z value of the vertex, we mark the vertex as **ForceSupport**.

#### Generate supporting areas

We add all grid points with the supporting type **ForceSupport**, hanging edges, and hanging points into the supporting areas. We calculate the angles between the normals of grid points with the supporting type **None** and the X-axis. If angles are greater than a threshold, then the grid points are added to the supporting areas. Lastly, we remove all the grid points with the supporting type **ForceNoSupport** from the supporting areas. Support structures are generated after the final supporting areas are obtained.

## Results

We conducted numerous experiments to test our slicing and support structure generation algorithm. This section shows some typical experimental results.

### Model slicing

**Fig. 7:**
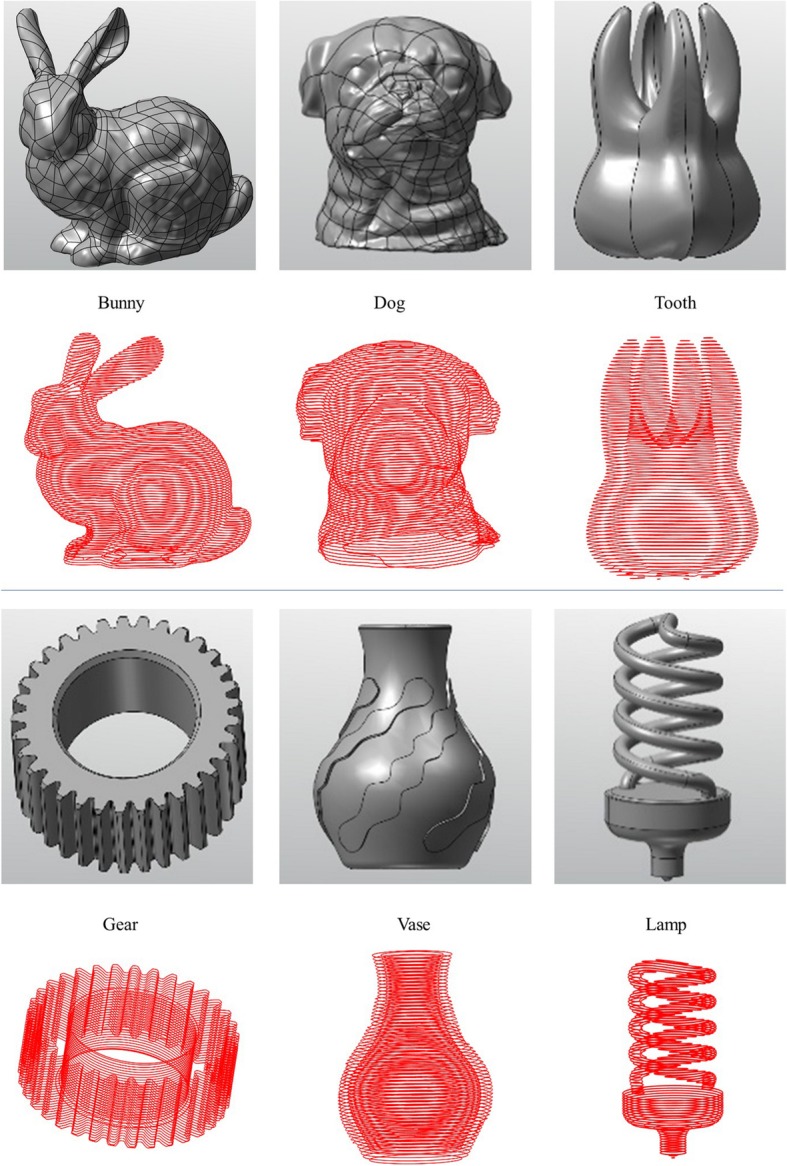
Slicing result of different B-rep models

The experimental results of the six models are presented in Fig. 7 and Table 1. As shown in Fig. 7, the gray models are six B-rep models, and the red sliced models are the slice results of the respective B-rep models above them by our model slicing algorithm. Table 1 shows the face and slice numbers of these six models. The third column in the table shows that these B-rep models have different face types. It proves that our model slicing algorithm can handle different face types in different B-rep models.

**Table 1:** Time cost of different B-rep models

Model	Face	Face type		Slice	Time
Bunny	700	B-Spline	700	425	1.464
Dog	322	B-Spline	322	378	3.588
Tooth	8	B-Spline	8	343	1.153
Gear	206	Plane	2	260	1.082
		Cylinder	68		
		Cone	70		
		B-Spline	66		
Vase	43	Plane	3	606	5.645
		Torus	6		
		B-Spline	20		
		Rational B-spline	14		
Lamp	23	Plane	3	635	2.732
		Cylinder	4		
		Sphere	2		
		Rational B-spline	14		

The experimental results of the six models are presented in Fig. 7 and Table 2. We compare our algorithm with the algorithms in Slic3r [16] and Cura [15]. Our algorithm works directly on the B-rep models, whereas Slic3r and Cura work on STL models converted from the same B-rep models. For a fair comparison, we require the same 3D printing precision, which is 0.1 mm, and the same number of layers. According to Table 2, our algorithm consumes considerably less time than Slic3r and Cura.

**Table 2:** Time cost of different algorithm

Model	Time		
	Ours	Slic3r	Cura
Finger Plate	0.037 s	5.239 s	0.124 s
Wristband	0.180 s	15.323 s	0.484 s
Christmas tree	0.020 s	1.958 s	0.031 s
Cup	0.128 s	14.044 s	0.671 s
Pen Holder	0.271 s	35.633 s	1.607 s
Bowl	0.022 s	1.893 s	0.047 s

### Support structure generation

**Fig. 8:**
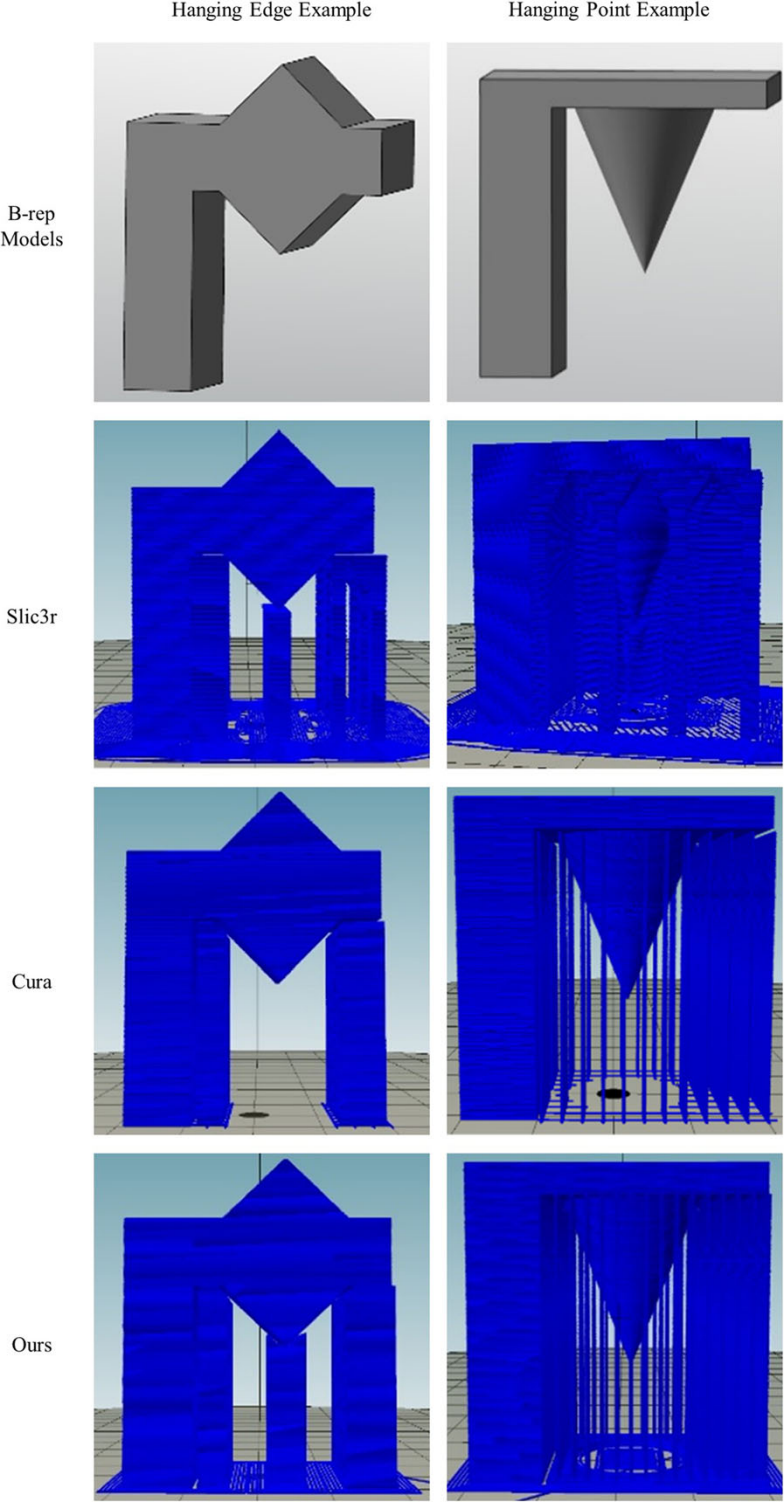
Examples of supporting structure generation with different methods

In this study, the support generation algorithm generates the support of the model hanging edge and the suspension point. One way to accomplish this is to automatically generate the corresponding support at the bottom of the suspension edge and the suspension point by detecting the suspension edge and the suspension point. In this study, we first used the Repetier-Host software to visualize the model support generation. The support generation algorithm in this study is shown in Fig. 8. The left and right in Fig. 8 are the hanging edge and point examples with the support generation results of Slic3r, Cura, and our support generation algorithm, respectively. Slic3r is generated by default support, and the Cura support generated suspension angle is set to π/3, the same as with our detecting algorithm. The support area fill types are both set to a straight-line fill. As shown in Fig. 8, Slic3r can detect a hanging edge well, but cannot handle a hanging point well. The support generated by Slic3r for the hanging point example is very redundant. Cure can detect a hanging point well, but cannot detect a hanging edge. Our algorithm can detect both the hanging edge and the hanging point in the model, and the support structures are well-generated, with almost no redundancy.

**Fig. 9:**
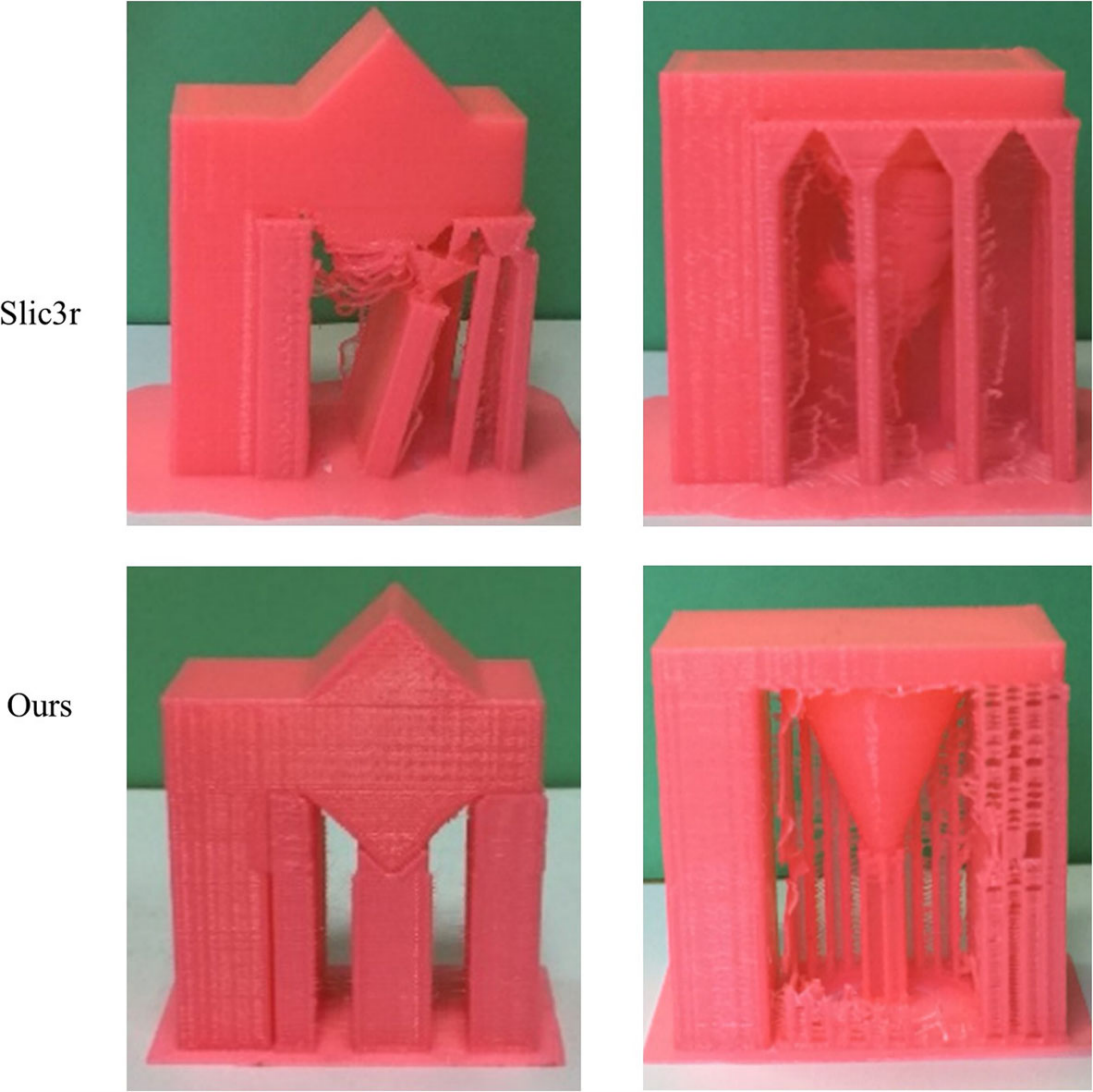
Print result of supporting structure generation with different methods

The examples shown in Fig. 8 prove that our algorithm can generate support structures well for hanging edges and points. After the step of support generation, we print the two examples in Fig. 8 with Slic3r and with our algorithms, respectively. The comparison of print results with our algorithm and the Slic3r is shown in Fig. 9. For the two examples, the support structures generated by Slic3r cannot support 3D printing well. Some parts of the models fail to be printed. By comparison, our algorithm can work well on the two examples. The triangle and the cone in the middle of the models are well-printed.

## Discussion and conclusion

This paper proposes a slicing and support structure generation algorithm that can work directly on B-rep models. The experimental results show that our algorithm can improve the efficiency and stability of 3D printing. Our algorithm also allows users to specify the supporting types, thus increasing the flexibility of 3D printing.

In the future, we will continue to improve the efficiency of 3D printing. We will attempt to find some new ways of reducing the cost of support structures while retaining the stability.

## Abbreviations

B-Rep: Boundary representation
CAD: Computer aided design
FDM: Fused deposition modelling
NURBS: Non-Uniform Rational B-Splines
STEP: Standard for the exchange of product model data
STL: Stereo lithography

## Authors’ information

Kanle Shi is currently an assistant professor in School of Software at Tsinghua University. His research interests include 3D printing, CAD and computer graphics.

Conghui Cai is currently a master candidate in School of Software at Tsinghua University. His research interests include CAD and computer graphics.

Zijian Wu is currently an engineer in School of Software at Tsinghua University. His research interests are CAD and computer graphics.

Junhai Yong is a professor in School of Software at Tsinghua University. His main research interests include CAD and computer graphics.
